# Safety and antibody responses to inactivated COVID-19 vaccines among elderly patients with COPD: a prospective cohort study

**DOI:** 10.3389/fimmu.2026.1840305

**Published:** 2026-06-11

**Authors:** Xuan Deng, Lijuan Xu, Lifeng Chen, Pinjing Sun, Hexiang Jia, Renping Shu, Zheren Wu, Hangjie Zhang, Xiaoping Xu

**Affiliations:** 1 Department of Immunization Program, Zhejiang Provincial Center for Disease Control and Prevention, Hangzhou, China; 2 Department of Epidemiology and Health Statistics, School of Public Health, Wuhan University, Wuhan, China; 3 Yuyao Municipal Center for Disease Control and Prevention, Ningbo, China; 4 Haining Municipal Center for Disease Control and Prevention, Jiaxing, China; 5 Xiaoshan District Center for Disease Control and Prevention, Hangzhou, China; 6 Jiande Municipal Center for Disease Control and Prevention, Hangzhou, China; 7 Department of Prevention and Control of Infectious Disease, Key Lab of Vaccine, Zhejiang Provincial Center for Disease Control and Prevention, Hangzhou, China; 8 School of Public Health, Hangzhou Medical College, Hangzhou, China

**Keywords:** COPD, COVID-19, immunogenicity, safety, vaccine

## Abstract

**Background:**

Elderly patients with chronic obstructive pulmonary disease (COPD) are at increased risk of severe coronavirus disease 2019 (COVID-19) outcomes. We aimed to assess the safety and longitudinal antibody dynamics of a three-dose schedule of the inactivated COVID-19 vaccine (Vero cell, Covilo) in elderly patients with COPD.

**Methods:**

This prospective cohort study enrolled 410 COPD patients (aged≥60), 80 younger healthy controls (HCs, 18–59 years), and 108 older HCs (≥60 years) in Zhejiang Province, China. COPD patients received a three-dose regimen (Day 0, 21, and 111), while HCs received a standard two-dose series. Neutralizing antibodies (NAbs), anti-receptor-binding domain IgG (anti-RBD IgG), and anti-spike & nucleocapsid IgG (anti-S&N IgG) were measured at five time points to evaluate the immunogenicity. Safety was assessed within 7 days after each dose and throughout follow-up.

**Results:**

The overall incidence of adverse events in COPD patients was 10.24%, predominantly mild (Grade 1), with decreasing frequency across doses. After the two-dose primary series, NAbs seroconversion rates at 28–35 days were comparable between COPD patients (60.19%) and HCs (63.64%–66.67%). However, antibody concentration declined markedly within three months, particularly for NAbs. Strong positive correlations were observed among NAbs, anti-RBD IgG, and anti-S&N IgG (Spearman’s ρ = 0.754–0.905; p< 0.001). A third dose administered after approximately three months significantly increased NAbs seroconversion rate to 83.50% and markedly elevated antibody concentrations to 207.22 U/mL. Despite the booster, NAbs declined significantly six months after the third dose.

**Conclusions:**

A three-dose regimen of Covilo is safe and effectively elicits antibody responses in elderly COPD patients. However, the rapid waning of antibody levels suggests a need for optimized booster strategies to maintain long-term protection in this vulnerable group.

## 1 Introduction

The global pandemic of coronavirus disease 2019 (COVID-19) continues to pose a substantial public health crisis. According to the World Health Organization (WHO), as of February 1, 2026, more than 779 million confirmed cases and approximately 7.11 million deaths have been reported worldwide (1). Chronic obstructive pulmonary disease (COPD), a common chronic respiratory disorder (CRD) characterized by persistent airflow limitation and chronic airway inflammation, is a well-recognized risk factor for poor COVID-19 outcomes, as these patients exhibit impaired respiratory function and compromised immune status, making them more susceptible to SARS-CoV-2 infection and its severe sequelae (2–5). Therefore, optimizing vaccination strategies for this vulnerable population is of critical importance.

Evidence suggests that vaccination not only prevents acute infection but also alleviates symptoms such as fatigue and lung impairment and lowers the risk of hospitalization and respiratory failure in patients with CRD (6–8). Large-scale data from Italy demonstrated that mass vaccination significantly reduced COPD-related mortality, with a greater benefit observed in older adults (9). Nevertheless, COPD patients exhibit varying degrees of COVID-19 vaccine hesitancy, largely due to concerns about safety and immunogenicity (10–12). Strengthening evidence on vaccine safety and immunogenicity in this high-risk population is essential to improve vaccination uptake.

During the initial nationwide vaccination rollout in early 2021, a two-dose regimen of inactivated SARS-CoV-2 vaccines (Vero cell) were extensively administered in China (13) due to their favorable safety profile and proven immunogenicity in the general population (14). However, with the global surge of the Delta and Omicron variant in late-2021, breakthrough infections raised public concern about waning immunity (15, 16). Most existing studies (17, 18) have focused on healthy individuals or mixed chronic disease cohorts. Prospective data specifically addressing elderly COPD patients, particularly regarding longitudinal antibody dynamics and safety following primary series of inactivated vaccine, remain limited and inconclusive. Given the potential impact of immunosenescence and chronic inflammation on vaccine response and adverse event risks, dedicated evaluation in this high-risk population is warranted.

The Chinese government started to considering booster doses to enhance and sustain immunity, particularly in high-risk groups. From late 2021 to early 2022 in China, booster doses were initially prioritized for high-risk groups, including COPD patients (19). However, the effect of a booster dose of inactivated vaccine on antibody persistence in elderly COPD patients has not been fully elucidated, and the optimal timing for its administration has yet to be established. Additionally, further assessment remains warranted in elderly COPD patients to clarify how vaccine-induced antibodies—including neutralizing antibodies (NAbs), anti-receptor binding domain IgG (anti-RBD IgG) and anti-spike & nucleocapsid IgG (anti-S&N IgG)—correlate with one another and how durable they are following primary and booster immunization.

To address these gaps, we conducted a prospective cohort study in Zhejiang Province, China, to assess the safety and antibody response of the inactivated SARS-CoV-2 vaccine (Vero cell, Covilo) among elderly COPD patients aged ≥60 years, compared with younger and older healthy controls (HCs). By longitudinally analyzing antibody dynamics, this study aimed to provide evidence-based data to inform COVID-19 vaccination strategies for elderly COPD patients, especially to evaluate the persistence of the primary series and to explore the optimal booster vaccination strategies with Covilo in this vulnerable population.

## 2 Methods

### 2.1 Study design and participants

This non-randomized, single-center, open-label phase 4 clinical trial was conducted in Zhejiang Province, China, between August 12, 2021, and November 6, 2022. A total of 410 patients with COPD aged ≥60 years, 80 HCs aged 18–59 years (18–59 HCs), and 108 HCs aged ≥60 years (≥60 HCs) were enrolled. All participants received at least one dose of vaccine and completed at least one antibody assessment and safety evaluation. The study was approved by the Ethics Committee of Zhejiang Center for Disease Control and Prevention (approval number: 2021-026-01) and was prospectively registered with https://clinicaltrials.gov/ (NCT05104216). All participants provided written informed consent.

The inclusion criteria for the COPD group were: (1) age ≥60 years; (2) diagnosis of COPD according to the 2021 revised edition of the Chinese Guidelines for the Diagnosis and Treatment of Chronic Obstructive Pulmonary Disease; and (3) stable management of other chronic comorbidities. Exclusion criteria included: (1) prior SARS-CoV-2 infection; (2) previous COVID-19 vaccination; (3) known allergy to any vaccine component; (4) receipt of non-specific immunoglobulin within one month prior to enrollment; and (5) administration of a live attenuated vaccine within one month or any other vaccine within 14 days prior to enrollment.

The inclusion criteria for the HCs group were no diagnosis of COPD or any other known disease. Exclusion criteria for HCs groups were identical to those of the COPD group.

### 2.2 Sample size

The primary immunogenicity endpoint was the seroconversion rate (SCR) of NAbs at 28 days after the second dose. Sample size was calculated based on the formula for non-inferiority testing:


$$n_{c}=\frac{{\left(Z_{1-\alpha }+Z_{1-\beta }\right)}^{2}}{{\left(\left|\pi_{T}-\pi_{C}\right|-\Delta \right)}^{2}}\left[\frac{\pi_{T}\left(1-\pi_{T}\right)}{K}+\pi_{C}\left(1-\pi_{C}\right)\right]$$

Based on phase III data of the Beijing Institute of Biological Products, the SCR of NAbs elicited by Covilo was 82% in individuals aged ≥60 years. Therefore, the control group SCR (π_C_) was set at 0.82. Given the underlying health conditions of the elderly COPD population, an SCR (π_T_) of 0.70 was assumed. The non-inferiority margin (Δ) was set at 0.01, with a two-sided α of 0.05 and 80% power. Considering China’s COVID-19 vaccination rollout policy at the time of enrollment, a 2:1 allocation ratio was adopted between the intervention and control groups. Considering a 20% loss to follow-up, the target sample size for the COPD group was set at 400 participants.

### 2.3 Procedures

**Figure 1:**
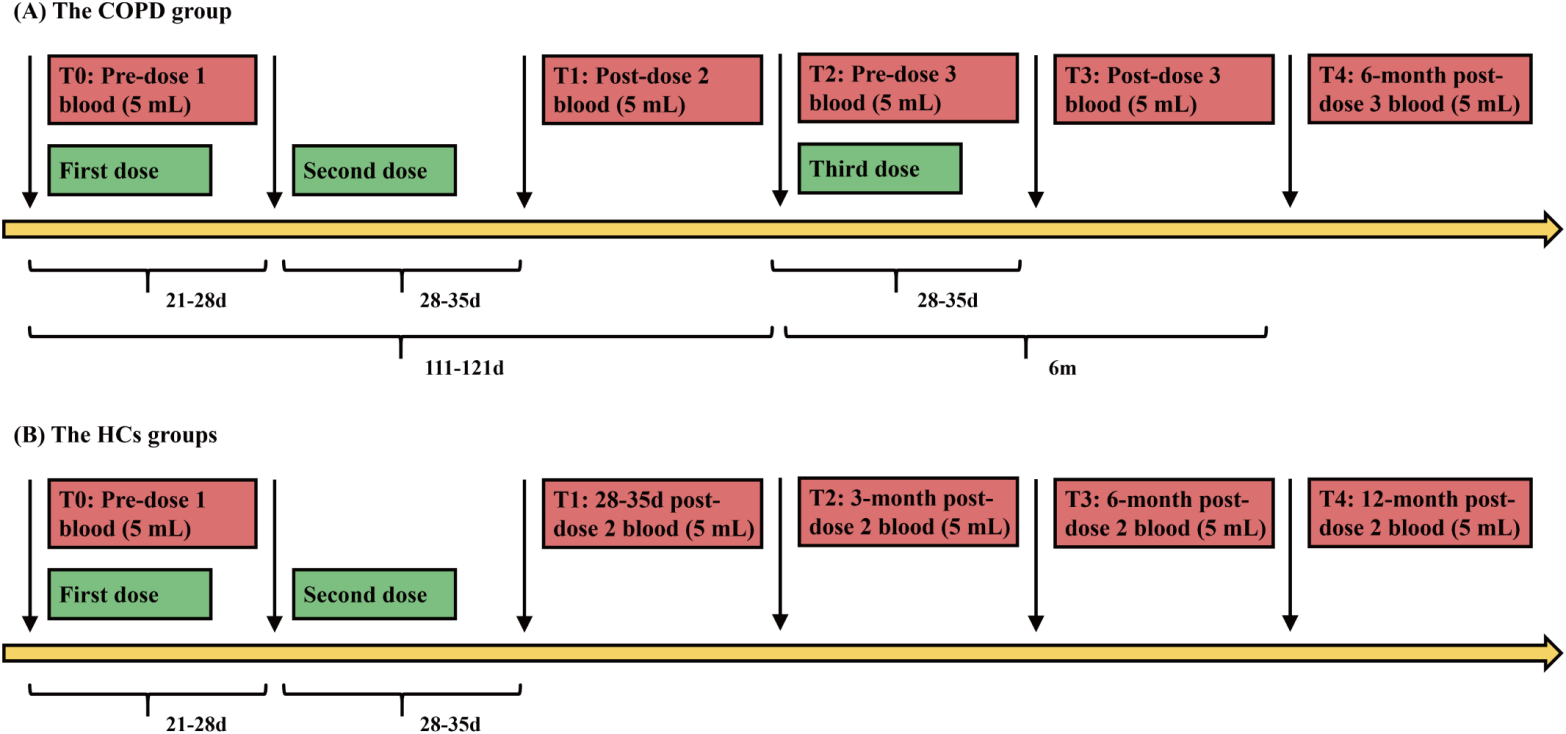
Vaccination and blood collection schedule.

The Covilo, produced by Beijing Institute of Biological Products Co., Ltd. (batch number: 2021020153), was transported at 2–8 °C. The vaccination regimen consisted of three intramuscular injections (0.5 mL each) into the deltoid muscle of the non-dominant arm, administered on day 0, day 21 (± 7 days), and day 111 (± 10 days). A minimum interval of 90 days was maintained between the second and third doses. Due to China’s COVID-19 vaccination policy then, third doses were restricted to high-risk populations only, thus the COPD group received all three doses, whereas the HCs groups received the standard two-dose primary series (**Figure 1**).

### 2.4 Safety assessment

Participants were observed for 30 minutes post-vaccination for immediate adverse reactions. Active safety monitoring was conducted on days 1, 3, 7, and 21 after each dose, and every 3 months after the last dose to record any solicited or unsolicited adverse events (AEs). Passive safety surveillance was conducted throughout the study period, and participants were instructed to report AEs spontaneously. AEs were graded according to the *Guidelines for Adverse Event Classification Standards for Clinical Trials of Preventive Vaccines (*20). Serious adverse events (SAEs) were monitored throughout the study period: for up to 6 months post-3rd dose for the COPD group and 12 months post-2nd dose for the HC groups.

### 2.5 Laboratory testing

Peripheral venous blood samples (5 mL) were collected at baseline (T0), 28–35 days (T1) and 3 months (T2) after the second dose, and at 28–35 days (T3) and 6 months (T4) after the third dose in the COPD group, or at 6 months (T3) and 12 months (T4) after the second dose in the HCs groups. Samples were stored at –20 °C and tested at the China Institute for the Identification of Pharmaceutical and Biological Products.

NAbs responses against SARS-CoV-2 were quantified in serum or plasma using a competitive enzyme-linked immunosorbent assay (ELISA) (Anti-SARS-CoV-2 Neutralizing Antibody ELISA Kit, DD3101; Vazyme Biotech, China), performed in accordance with the manufacturer’s instructions. The assay is based on the inhibition of binding between the viral RBD and the human angiotensin-converting enzyme 2 (ACE2) receptor. Briefly, diluted samples (1:10) and reference standards were pre-incubated with horseradish peroxidase (HRP)-conjugated RBD, and the mixtures were subsequently transferred to ACE2-coated microplates. Following incubation and washing, color development was achieved using tetramethylbenzidine substrate, and absorbance was measured at 450 nm. NAbs concentrations were calculated using a four-parameter logistic (4-PL) standard curve generated from serially diluted reference standards. A concentration of ≥10.0 U/mL was considered positive.

Total binding IgG levels were evaluated using two distinct commercial platforms (21, 22). Quantification of IgG antibodies specific to the SARS-CoV-2 spike (S) and nucleocapsid (N) proteins was performed using the iFlash 2019-nCoV kit (Shenzhen, China). Serum was initially incubated with paramagnetic microparticles coated with S and N antigens. An acridinium-ester–labeled angiotensin-converting enzyme 2 (ACE2) conjugate was subsequently introduced to compete for binding sites. The resulting relative light units (RLUs) were translated into antibody concentrations (AU/mL) via a two-point calibration system. Samples were considered positive if anti-S&N IgG concentrations ≥10.0 AU/mL. IgG against the RBD were measured using a specialized enzyme-linked immunosorbent assay (ELISA) kit (Bioscience Biotech, Chongqing, China). Following manufacturer protocols, a concentration of ≥1.0 AU/mL was defined as positive for anti-RBD IgG.

### 2.8 Outcomes

The primary safety endpoint was the incidence and severity of AEs within 7 days following each dose of vaccine.

The primary immunogenicity endpoint was the SCR of NAbs at T1. Major secondary immunogenicity endpoints included the seropositivity rate (SPR) and the geometric mean concentration (GMC) of NAbs at T2, SCR and geometric mean fold increase (GMFI) of NAbs at T3, and above indexes of anti-RBD IgG and anti-S&N IgG at corresponding timepoints, with corresponding 95% confidence intervals (CIs).

### 2.9 Statistical analysis

The safety analyses included all participants who received at least one dose of vaccine. The immunogenicity analyses included participants who completed the full immunization schedule (3 doses for COPD, 2 doses for HCs) and all five scheduled serological tests, as well as adherence to the protocol-defined window.

Categorical variables were presented as counts and percentages [n (%)], and continuous variables were expressed as mean ± standard deviation (SD) or geometric mean with 95% CI, depending on the distribution. The relationships among the three antibodies were assessed using Spearman’s rank correlation analysis. The Chi-square test or Fisher’s exact test was used to compare AEs, SCRs and SPRs, with the Bonferroni correction applied for pairwise comparisons. Levels of antibodies against SARS-CoV-2 are presented as the SCRs, GMCs, SPRs, and GMFIs with 95% CI. NAbs levels below the detection limit (1:10) were assigned a value of 5 U/mL (half of the threshold) to enable calculation of GMC and GMFI after logarithmic transformation. GMFI was calculated as the geometric mean of individual post-vaccination to baseline ratios (T1/T0). The 95% CI was calculated based on the t-distribution of the log-transformed values back-transformed to the original scale. The Kruskal-Wallis’s test followed by Dunn’s multiple comparisons was applied for analyzing GMCs and GMFIs. All analyses were conducted using GraphPad Prism (version 8.2.1), IBM SPSS Statistics (version 21.0) and R (version 4.4.1). A two-sided P-value<0.05 was considered statistically significant.

## 3 Results

### 3.1 Demographic characteristics

**Figure 2:**
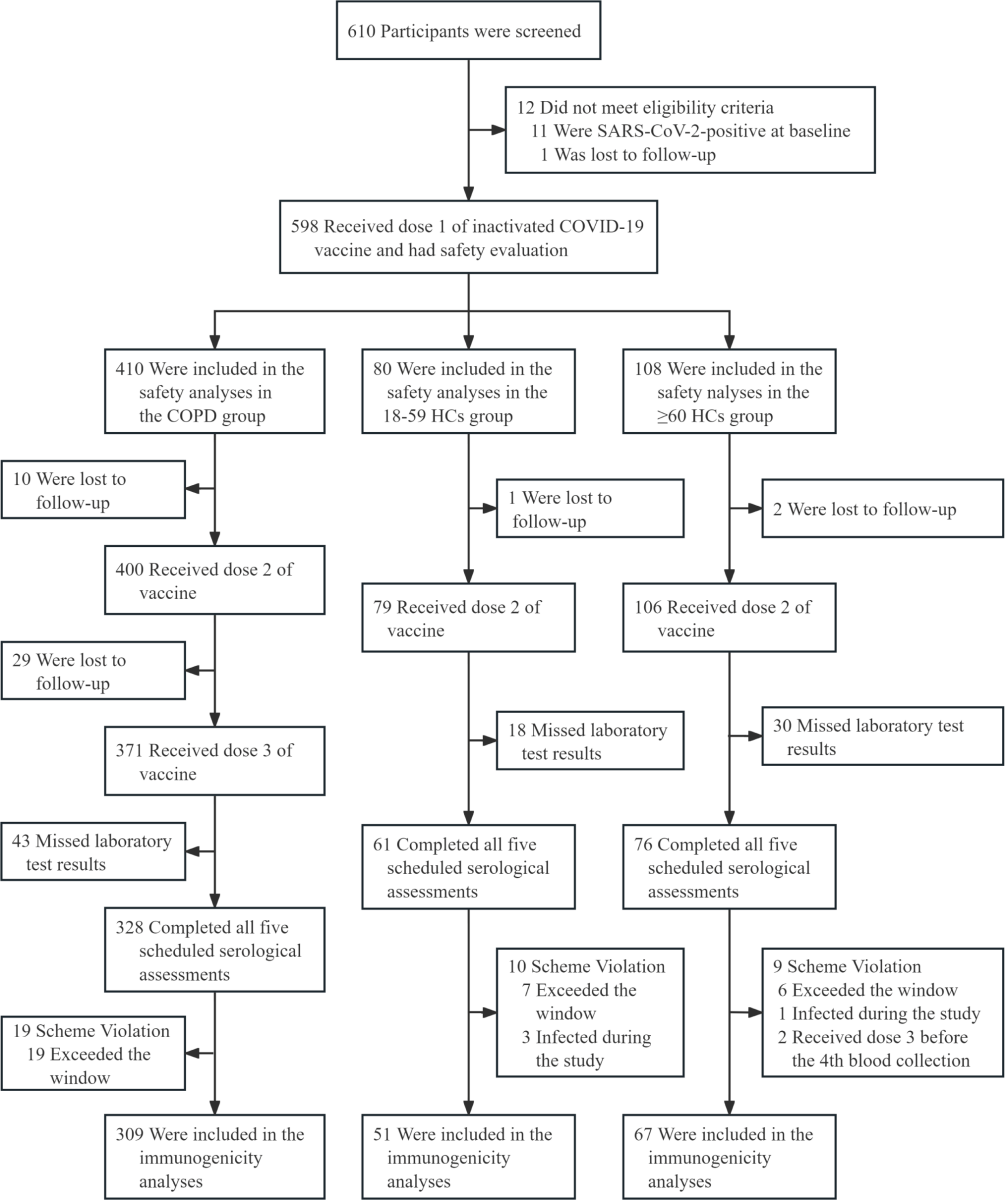
Visit completion status. The data cutoff for the primary analysis occurred on October 18, 2021. The full analysis population excluded individuals who met the exclusion criteria or were lost to follow-up. The safety population included participants who received at least one dose of Covilo and were observed at least one for safety outcomes. The immunogenicity analysis population comprised participants who completed the full immunization schedule (3 doses in the COPD group and 2 doses in both HCs groups) and completed all five scheduled serological laboratory assessments.

A total of 610 participants were recruited (**Figure 2**), with 598 eligible (98.03%) for safety analysis, including 410 COPD patients, 80 younger HCs (18–59 years), and 108 older HCs (≥60 years); and 427 eligible (71.40%) for immunogenicity analysis, including 309 COPD patients, 51 younger HCs, and 67 older HCs. Demographic characteristics were shown in **Table 1**. Among COPD patients, 236 (57.56%) were men, with mean (SD) age of 71 (6.92) years. Pulmonary function was predominantly grade I (38.54%), followed by grade II (27.32%), and grade III (21.95%).

**Table 1:** Baseline characteristics of the participants.

Characteristics	COPD (N = 410)	18–59 HCs (N = 80)	≥60 HCs (N = 108)
Sex			
Male	236 (57.56)	41 (51.25)	46 (42.59)
Female	174 (42.44)	39 (48.75)	62 (57.41)
Age (years)			
Mean ± SD	71.28 ± 6.92	44.29 ± 11.60	70.22 ± 6.71
Median	71	47	69
Min, Max	60, 91	24, 59	60, 90
Region			
Jiande	13 (3.17)	13 (16.25)	20 (18.52)
Xiaoshan	44 (10.73)	17 (21.25)	20 (18.52)
Haining	200 (48.78)	–	–
Yuyao	153 (37.32)	50 (62.50)	68 (62.96)
SBP (mmHg)			
Mean ± SD	137.06 ± 14.35	128.00 ± 11.41	131.80 ± 11.15
Median	138	128.5	130
Min, Max	92, 159	92, 158	106, 159
DBP (mmHg)			
Mean ± SD	78.24 ± 8.82	78.86 ± 8.05	78.01 ± 6.88
Median	80	80	78.5
Min, Max	54, 99	60, 98	60, 94
Pulmonary function			
Grade I	158 (38.54)		
Grade II	112 (27.32)		
Grade III	90 (21.95)		
Grade IV	50 (12.20)		

SBP, systolic blood pressure; DBP, diastolic blood pressure.

### 3.2 Safety

**Figure 3:**
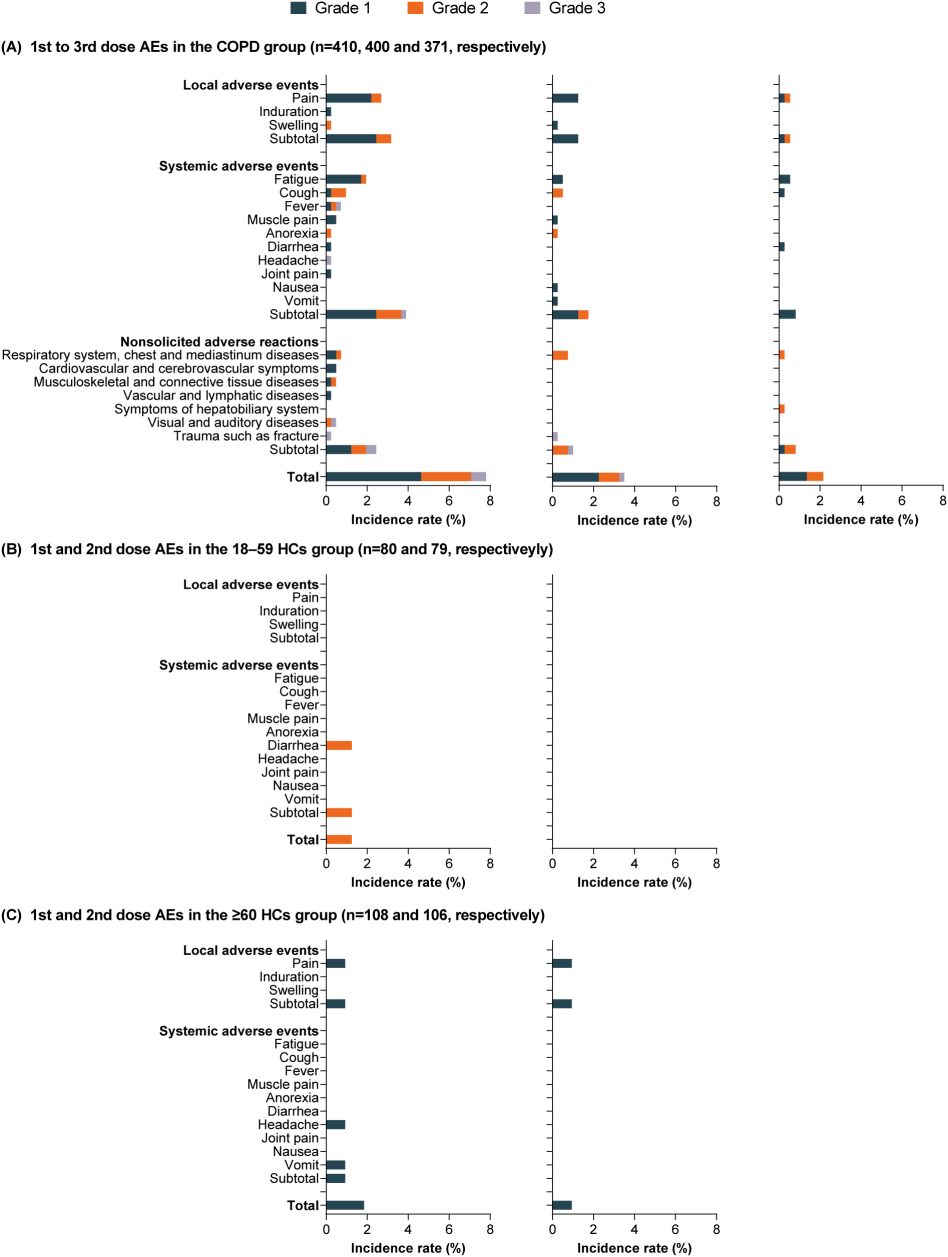
AEs that occurred during the study period after each dose of vaccination. **(A)** AEs following the first to third vaccine doses in the COPD group (n = 410, 400, and 371, respectively). **(B)** AEs following the first and second vaccine doses in the 18–59 HCs group (n = 80 and 79, respectively). **(C)** AEs following the first and second vaccine doses in the ≥60 HCs group (n = 108 and 106, respectively). Data on local and systemic reactions and any unsolicited events were collected with electronic diaries from 598 participants throughout the study in the safety subset after each vaccination. No unsolicited events were reported in the HCs groups. AEs were categorized by severity as Grade 1 (dark blue), Grade 2 (orange), and Grade 3 (grey).

The incidence of AEs within 7 days of each dose were summarized in **Table 2**. Overall, 42 cases reported AEs (10.24%) in COPD patients and the AE rates after each dose were 6.34%, 2.50% and 1.62%, respectively. No vaccine-related SAE was reported throughout the study period (**Supplementary Table 1**). The most frequently reported local and systemic AEs were respectively pain at the injection site (2.68%, 1.00%, 0.54% after each dose), and fatigue/weakness (1.95%, 0.50%, 0.54% after each dose), most of which were Grade 1 on severity (**Figure 3**). A significant decreasing trend was observed across doses for overall AEs ( $\chi_{trend}^{2}=12.944$, *p* < 0.001), local AEs ( $\chi_{trend}^{2}=8.710$, *p* = 0.003) and systematic AEs ( $\chi_{trend}^{2}=6.028$, *p* = 0.014). The safety outcomes were similar in HCs, with the overall AEs of 1.25% for 18–59 years old and 3.70% for the elderly respectively. For the 1st dose, though differences were detected for the overall AEs within 3 days (*p* = 0.042) or 7 days (*p* = 0.042) among the three groups, no statistically significant differences were observed in pairwise comparisons (all *p* > 0.017; **Supplementary Table 2**). For the 2nd dose, the overall AE rates showed no intergroup statistically differences within 3 days or 7 days post-vaccination. In addition, AE severity distributions were similar across three groups (all *p* > 0.05) for both doses (details in **Supplementary Table 2**).

**Table 2:** AEs within 7 days after each dose of vaccination in the COPD group.

AEs	Dose 1 (n=410)	Dose 2 (n=400)	Dose 3 (n=371)	χ²/Fisher	*P* value
Local AEs					
Pain	11 (2.68)	4 (1.00)	2 (0.54)	7.133	**0.028**
Induration	1 (0.24)	0 (0.00)	0 (0.00)	–	1.000
Swelling	1 (0.24)	0 (0.00)	0 (0.00)	–	1.000
Rash	0 (0.00)	0 (0.00)	0 (0.00)	/	/
Redness/flush	0 (0.00)	0 (0.00)	0 (0.00)	/	/
Pruritus	0 (0.00)	0 (0.00)	0 (0.00)	/	/
Erythema	0 (0.00)	0 (0.00)	0 (0.00)	/	/
Subtotal	13 (3.17)	4 (1.00)	2 (0.54)	9.938	**0.007**
Systemic AEs					
Fatigue/weakness	8 (1.95)	2 (0.50)	2 (0.54)	–	0.088
Cough	3 (0.73)	2 (0.50)	1 (0.27)	–	0.877
Fever (axillary temperature)	3 (0.73)	0 (0.00)	0 (0.00)	–	0.111
Muscle pain (non-inoculated site)	2 (0.49)	1 (0.25)	0 (0.00)	–	0.778
Anorexia	1 (0.24)	1 (0.25)	0 (0.00)	–	1.000
Diarrhea	1 (0.24)	0 (0.00)	1 (0.27)	–	0.765
Headache	1 (0.24)	0 (0.00)	0 (0.00)	–	1.000
Joint pain	1 (0.24)	0 (0.00)	0 (0.00)	–	1.000
Nausea	0 (0.00)	1 (0.25)	0 (0.00)	–	0.653
Vomit	0 (0.00)	1 (0.25)	0 (0.00)	–	0.653
Constipation	0 (0.00)	0 (0.00)	0 (0.00)	/	/
Dysphagia	0 (0.00)	0 (0.00)	0 (0.00)	/	/
Dyspnea	0 (0.00)	0 (0.00)	0 (0.00)	/	/
Non-vaccination site pruritus (no skin damage)	0 (0.00)	0 (0.00)	0 (0.00)	/	/
Dermatomycosis abnormalities	0 (0.00)	0 (0.00)	0 (0.00)	/	/
Acute anaphylaxis	0 (0.00)	0 (0.00)	0 (0.00)	/	/
Subtotal	13 (3.17)	6 (1.50)	3 (0.81)	6.380	**0.041**
Non-solicited adverse reactions					
Respiratory system, chest and mediastinum diseases	2 (0.49)	1 (0.25)	0 (0.00)	–	0.778
Cardiovascular and cerebrovascular symptoms	2 (0.49)	0 (0.00)	0 (0.00)	–	0.333
Musculoskeletal and connective tissue diseases	1 (0.24)	0 (0.00)	0 (0.00)	–	1.000
Vascular and lymphatic diseases	1 (0.24)	0 (0.00)	0 (0.00)	–	1.000
Symptoms of hepatobiliary system	0 (0.00)	0 (0.00)	1 (0.27)	–	0.314
Visual and auditory diseases	0 (0.00)	0 (0.00)	0 (0.00)	/	/
Trauma such as fracture	0 (0.00)	0 (0.00)	0 (0.00)	/	/
Subtotal	6 (1.46)	1 (0.25)	1 (0.27)	–	0.108
**Total**	26 (6.34)	10 (2.50)	6 (1.62)	14.641	**0.001**

SAEs are not included. The incidence of AEs (%) is calculated as (number of participants with at least one AE/total vaccinated participants) × 100%. Bold values indicate statistically significant differences (p < 0.05).

### 3.3 Immunogenicity

**Figure 4:**
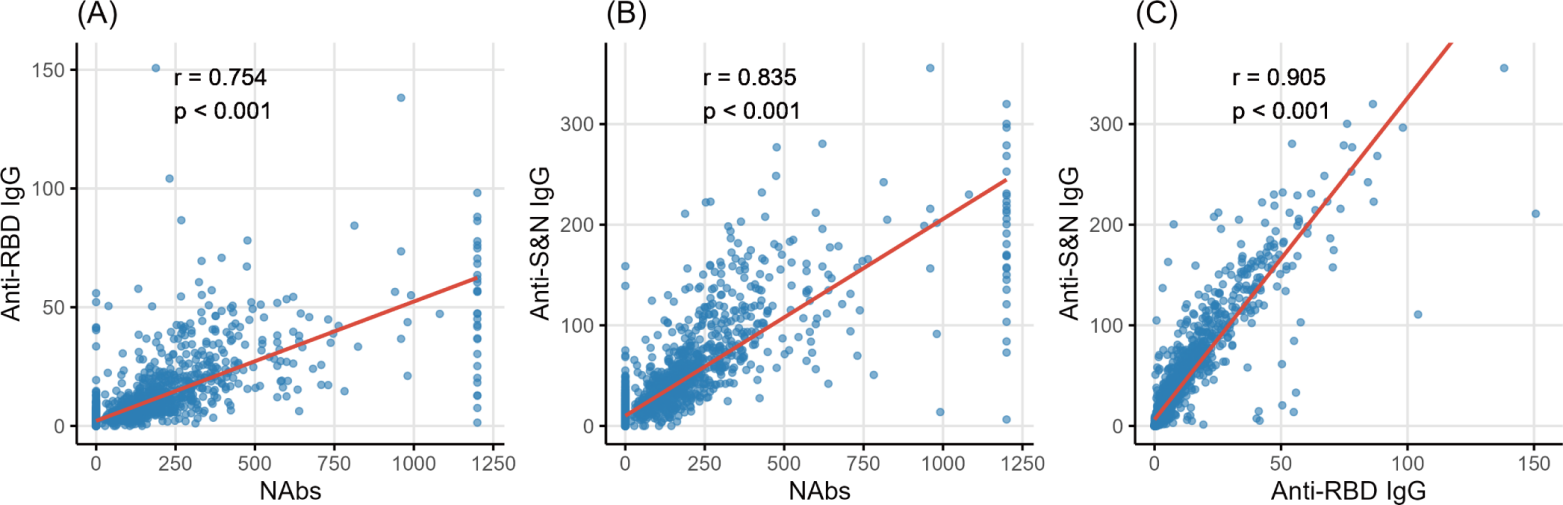
Correlation analysis of NAbs, anti-RBD IgG and anti-S&N IgG. **(A)** Correlation between NAbs and anti-RBD IgG. **(B)** Correlation between NAbs and anti-S&N IgG. **(C)** Correlation between anti-RBD IgG and anti-S&N IgG.

As shown in **Figure 4**, strong positive correlations were observed among levels of NAbs, anti-RBD IgG, and anti-S&N IgG (Spearman’s ρ = 0.754–0.905, *p* < 0.001), suggesting coordinated humoral responses across different assay platforms.

#### 3.3.1 Primary immunization

**Figure 5:**
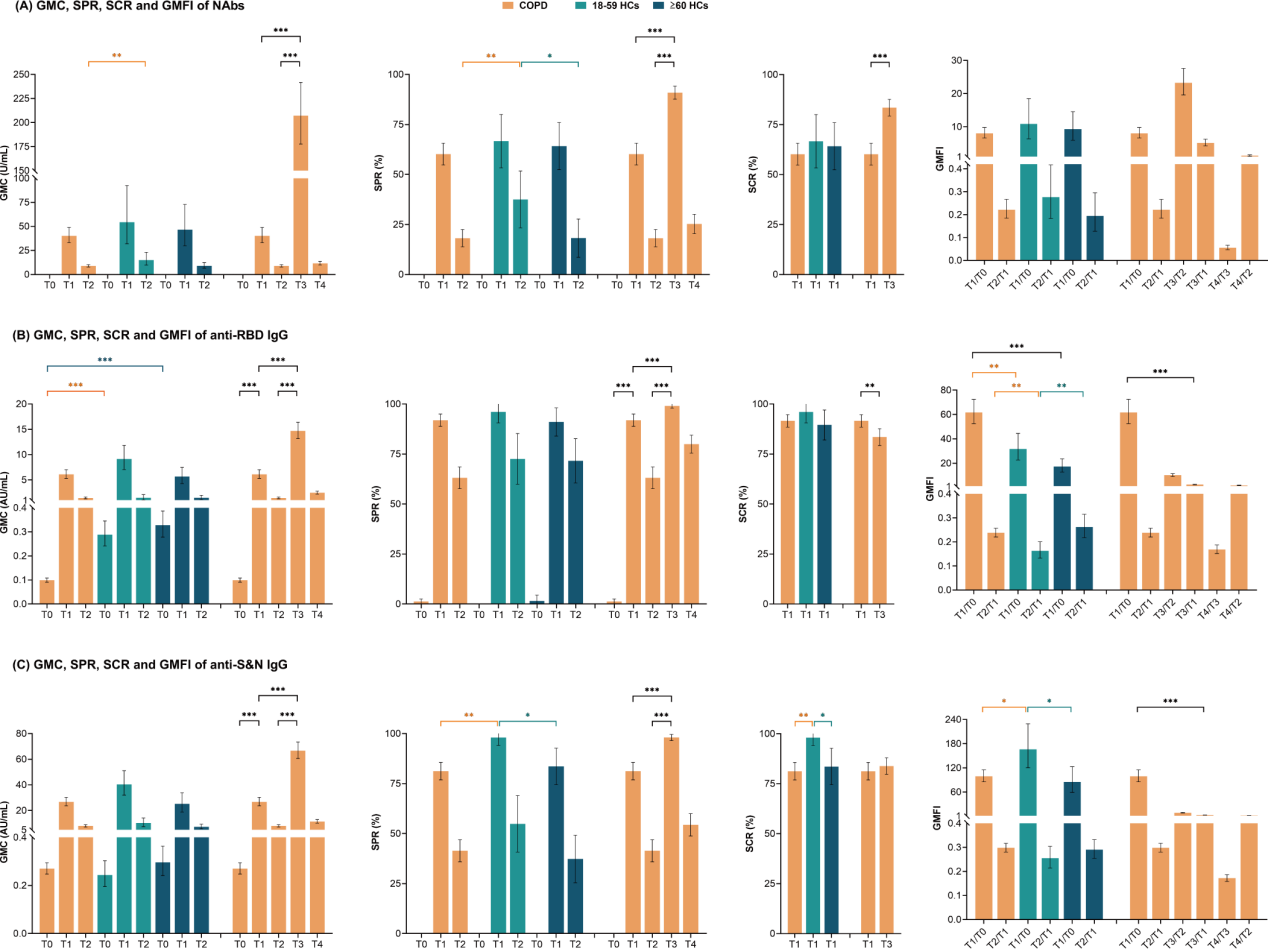
NAbs, anti-RBD IgG and anti-S&N IgG. **(A)** GMC, SPR, SCR, and GMFI of of NAbs. **(B)** GMC, SPR, SCR, and GMFI of anti-RBD IgG. **(C)** GMC, SPR, SCR, and GMFI of anti-S&N IgG. T0, before the 1st dose; T1, 28–35 days after the 2nd dose; T2, 3 months after the 2nd dose; T3, 28–35 days after the 3rd dose (COPD group) or 6 months after the 2nd dose (HCs groups); T4, 6 months after the 3rd dose (COPD group) or 12 months after the 2nd dose (HCs groups). Group colors: COPD (orange), 18–59 HCs (blue-green), and ≥60 HCs (dark blue).

As illustrated in **Figure 5** and **Table 3**, all participants were sero-negative for NAbs and anti-S&N IgG at baseline (T0). At T1, the SCRs of NAbs were 60.19%, 66.67% and 63.64% in the COPD group, the 18–59 HCs group, and the ≥60 HCs group, respectively, with no significant intergroup differences (*p* > 0.05). The SCRs of anti-RBD IgG were respectively 91.59%, 96.08% and 89.55%, respectively, with similar intergroup distributions (*p* > 0.05). For anti-S&N IgG, the SCR in the COPD group was 81.23%, similar to 83.58% of the ≥60 HCs group (*p* > 0.05), but significantly lower than that in the 18–59 HCs group (98.04%, *p* < 0.05).

**Table 3:** NAbs, anti-RBD IgG and anti-S&N IgG.

Variables	COPD (n=309)					18–59 HCs (n=51)			≥60 HCs (n=67)		
	T0	T1	T2	T3	T4	T0	T1	T2	T0	T1	T2
NAbs											
GMC (U/mL)	0	40.17 (32.99, 48.92)	8.91 (7.74, 10.25)	207.22 (177.61, 241.78)	11.57 (9.80, 13.66)	0	54.32 (31.91, 92.48)	15.01 (9.75, 23.10)	0	46.55 (29.74, 72.87)	9.04 (6.59, 12.41)
SPR (%)	0.00 (0.00, 1.23)	60.19 (54.64, 65.49)	18.12 (14.23, 22.80)	90.94 (87.22, 93.66)	25.24 (20.72, 30.37)	0.00 (0.00, 7.41)	66.67 (52.54, 78.32)	37.50 (25.22, 51.64)	0.00 (0.00, 5.50)	63.64 (51.58, 74.19)	18.18 (10.72, 29.15)
SCR (%)	NA	60.19 (54.64, 65.49)	NA	83.50 (78.95, 87.22)	NA	NA	66.67 (78.32, 52.54)	NA	NA	63.64 (51.72, 75.55)	NA
Anti-RBD IgG											
GMC (AU/mL)	0.10 (0.09, 0.11)	6.11 (5.30, 7.04)	1.45 (1.28, 1.65)	14.71 (13.18, 16.41)	2.48 (2.20, 2.79)	0.29 (0.24, 0.35)	9.14 (7.04, 11.86)	1.49 (1.05, 2.10)	0.33 (0.28, 0.39)	5.67 (4.29, 7.48)	1.49 (1.11, 1.99)
SPR (%)	1.29 (0.50, 3.28)	91.91 (88.33, 94.46)	63.11 (57.60, 68.30)	99.03 (97.18, 99.74)	79.94 (75.11, 84.02)	0.00 (0.00, 7.00)	96.08 (86.78, 99.30)	72.55 (59.05, 82.89)	1.49 (0.08, 7.98)	91.04 (81.81, 95.83)	71.64 (59.91, 81.03)
SCR (%)	NA	91.59 (87.96, 94.19)	NA	83.50 (78.95, 87.22)	NA	NA	96.08 (86.78, 99.30)	NA	NA	89.55 (79.97, 94.85)	NA
Anti-S&N IgG											
GMC (AU/mL)	0.27 (0.25, 0.29)	26.77 (23.65, 30.29)	7.98 (7.11, 8.97)	66.67 (60.61, 73.34)	11.47 (10.18, 12.92)	0.24 (0.20, 0.30)	40.40 (31.98, 51.02)	10.30 (7.51, 14.13)	0.30 (0.24, 0.36)	25.21 (18.76, 33.89)	7.31 (5.70, 9.38)
SPR (%)	0.00 (0.00, 1.23)	81.23 (76.50, 85.19)	41.42 (36.07, 46.99)	98.06 (95.83, 99.11)	54.37 (48.80, 59.83)	0.00 (0.00, 7.00)	98.04 (89.70, 99.90)	54.90 (41.38, 67.73)	0.00 (0.00, 5.42)	83.58 (72.94, 90.58)	37.31 (26.72, 49.28)
SCR (%)	NA	81.23 (76.50, 85.19)	NA	83.82 (79.30, 87.51)	NA	NA	98.04 (89.70, 99.90)	NA	NA	83.58 (72.94, 90.58)	NA

NA, not applicable. Seropositivity is defined as follows: (1) a concentration of NAbs >10.0 U/mL; (2) a concentration of anti-RBD IgG ≥1.00 AU/mL; and (3) a concentration of anti-S&N IgG ≥10.00 AU/mL. Seroconversion is defined as either (1) a transition from a seronegative to a seropositive status after vaccination or (2) a 4-fold increase in antibody concentration in those who are positive before vaccination. The SPR (%) is calculated as (number of seropositive participants/total participants) × 100%, and the SCR (%) as (number of seroconverted participants/total participants) × 100%.

At T1, comparable GMCs and SPRs for NAbs and anti-RBD IgG were observed among the three groups. The GMCs of NAbs were 40.17, 54.32 and 46.55 U/mL respectively. Significantly higher SPR was observed in younger HCs (98.04%) versus COPD patients (81.23%, *p* = 0.003) and elderly HCs (83.58%, *p* = 0.010).

At T2, although the GMC of NAbs declined sharply to 8.91, the 18–59 HCs group showed a significantly higher GMC than the other two groups (P < 0.05). A similar trend was observed in SPR. The GMFIs (T2/T1) were 0.22, 0.28 and 0.19, respectively (details in **Table 4**). The comparable GMCs and SPRs for anti-RBD IgG and anti-S&N IgG were observed among the three groups.

**Table 4:** The GMFI of NAbs, anti-RBD and anti-S&N IgG.

Variables	COPD (n=309)						18–59 HCs (n=51)		≥60 HCs (n=67)	
	T1/T0	T2/T1	T3/T2	T3/T1	T4/T3	T4/T2	T1/T0	T2/T1	T1/T0	T2/T1
NAbs	8.04 (6.60, 9.78)	0.22 (0.18, 0.27)	23.26 (19.61, 27.60)	5.16 (4.26, 6.25)	0.06 (0.05, 0.07)	1.30 (1.09, 1.54)	10.86 (6.38, 18.50)	0.28 (0.18, 0.42)	9.31 (5.95, 14.57)	0.19 (0.13, 0.30)
Anti-RBD IgG	61.64 (52.44, 72.44)	0.24 (0.22, 0.26)	10.15 (9.02, 11.41)	2.41 (2.12, 2.74)	0.17 (0.15, 0.19)	1.71 (1.49, 1.97)	31.67 (22.51, 44.56)	0.16 (0.13, 0.20)	17.28 (12.66, 23.61)	0.26 (0.22, 0.32)
Anti-S&N IgG	99.18 (85.51, 115.00)	0.30 (0.28, 0.32)	8.35 (7.54, 9.26)	2.49 (2.25, 2.76)	0.17 (0.16, 0.19)	1.44 (1.28, 1.61)	166.00 (120.20, 229.40)	0.26 (0.21, 0.31)	85.12 (58.93, 123.00)	0.29 (0.25, 0.33)

All three groups exhibited similar patterns of antibody changes over different time points. However, the GMFIs (T2/T1) for the three antibodies differed significantly, with NAbs declining most rapidly, followed by anti-RBD IgG and then anti-S&N IgG (all *p* < 0.001). This indicates that NAbs possess the poorest durability within three months following primary immunization.

#### 3.3.2 Booster immunization

At T3, the SCR of NAbs increased to 83.50%, markedly exceeding the T1 level (60.19%, *p* < 0.05). The GMC of NAbs reached 207.22 U/mL, significantly higher than the level at T1 (*p* < 0.05). A similar increase was observed in the SPR, which rose to 83.50% (*p* < 0.05). Following the booster dose, antibody levels declined over time. At T4, the GMCs of three antibodies remained significantly higher than those at T2 (*p* < 0.05), and the SPR also showed a similar persistence.

The GMFI (T4/T3) of NAbs was significantly lower than that of the other two antibodies (*p* < 0.001), while no significant difference was observed between anti-RBD IgG and anti-S&N IgG (*p* > 0.05).

## 4 Discussion

To date, the COVID-19 pandemic continues to pose a substantial threat to immunocompromised populations worldwide (1). This prospective cohort study evaluated the safety and immunogenicity of a three-dose schedule of Covilo among COPD patients aged ≥60 years, finding that the vaccine was well tolerated in this population and elicited a modest antibody response against the wild-type SARS-CoV-2 strain following the primary two-dose series; however, antibody levels, particularly NAbs, declined rapidly within three months. A booster dose of Covilo administered at a three-month interval significantly enhanced antibody concentrations and SCRs. However, the durability of NAbs responses induced by the homologous booster did not persist for six months, highlighting the importance of optimizing booster strategies among elderly COPD patients.

In terms of safety, the overall incidence of AEs was 10.24%, predominantly mild (Grade 1), and decreased significantly across successive doses. The incidence of AEs after each dose among COPD patients was comparable to that among HCs. Consistent with previous findings among CRD populations, whose AE rates within one week after each dose of Covilo or CoronaVac were similar to healthy individuals (14.3% vs. 7.6%, *p* > 0.05) (23). A domestic self-controlled case series study also demonstrated that the incidence of respiratory AEs within 28 days after CoronaVac or BNT162b2 vaccination among COPD patients did not exceed the baseline levels observed prior to vaccination (24). In our study, the most common AEs were mild to moderate injection-site pain and fatigue, and AEs post the 1st dose were more frequent than that post the booster, which was align with population-based analyses (24) and studies in chronic pulmonary disease cohorts (25), which showed no increased risk of severe respiratory complications attributable to COVID-19 vaccination (26). Further suggesting good tolerability of repeated antigen exposure in this vulnerable population.

In terms of immunogenicity following the primary series, our study comprehensively evaluated humoral immune responses to Covilo and demonstrated strong positive correlations (Spearman’s ρ = 0.754–0.905) among the three types of antibodies measured using different assay methods. These findings are consistent with previous reports showing coordinated humoral responses after SARS-CoV-2 vaccination (27–29). Notably, a stronger correlation was observed between anti-S&N IgG and NAbs, suggesting that binding antibodies targeting S and N proteins may partially reflect functional neutralizing activity. Given that live virus–based neutralization assays are technically demanding, require biosafety level-3 facilities, anti-S&N IgG may serve as a practical alternative surrogate serological marker to estimate vaccine-induced humoral immunity to a certain extent (21).

Our study indicated that the SCRs of NAbs at T1 were comparable among three groups, which was consistent with the findings from Denmark with BNT162b2 (30) and Chongqing Province of China with inactivated vaccines (29). Though the induced NAbs level at T1 was comparable to HCs, the durability was significantly inferior to 18–59 HCs at T2, suggesting that primary series of inactivated COVID-19 vaccines only provided limited short-term protection for COPD patients. The findings were consistent with another Chinese study carried in 2021–2022 which exhibited significantly lower antibody titers (anti-RBD IgG: 2.10 vs. 3.45, *p* = 0.003; NAbs: 0.20 vs. 0.25, *p* = 0.007) and SPRs (anti-RBD IgG: 69.60% vs. 88.80%, *p* = 0.003; NAbs: 62.50% vs.79.70%, *p* = 0.011) among CRD patients after two doses of BBIBP-CorV or CoronaVac compared with healthy individuals (23). Similar waning immunity trend have been detected in studies of other COVID-19 vaccines developed using different technological platforms (31, 32). A study (33) focused on the deep immune phenotyping of humoral and cell-mediated responses to COVID-19 vaccines demonstrated that COPD patients exhibited significantly reduced anti-RBD IgG titers (*p* < 0.022) and decreased vaccine-specific memory CD8+ T-cells (*p* < 0.008) compared to HCs, suggesting both impaired humoral and cellular immunity in COPD patients following COVID-19 vaccination. This pattern is consistent with immunosenescence and chronic inflammation in older adults, which are known to impair vaccine responsiveness (29, 30, 34–38).

Encouragingly, administration of a booster dose markedly improved immunogenicity. COPD patients exhibited a rapid and marked increase for all the three types of antibodies following the booster dose. At T3, the SCR of NAbs increased to 83.50%, and GMC significantly exceeded those observed after the 2nd primary dose. This robust recall response suggests effective immune memory formation despite reduced initial responsiveness. Similar booster-induced enhancements have been reported for inactivated vaccines (39) and across other vaccine platforms (40, 41). Although antibody levels declined again over time (T4), they remained significantly higher than those observed after primary series (T2) except for NAbs, supporting the role of booster vaccination in prolonging humoral protection. These findings are particularly relevant for COPD patients, who are at increased risk of severe COVID-19 outcomes (42) and may exhibit attenuated baseline immune responses (23). Our data reinforce current recommendations advocating booster vaccination for COPD patients.

Nevertheless, the durability of antibody responses induced by homologous boosting with inactivated vaccines appears to be limited. Traditional randomized controlled trials (43, 44) have shown that the heterologous booster strategies, such as those using mRNA or adenovirus-vectored vaccines, elicited markedly higher binding and NAbs titers, along with stronger memory B-cell responses, compared with the homologous inactivated booster. Furthermore, an increasing number of real-world studies (45, 46) based on large-scale cohort have confirmed that a heterologous COVID-19 booster vaccination strategy provides superior protection against severe COVID-19 outcomes, particularly among populations with immunodeficiency. This pattern remains consistent among individuals primed with mRNA vaccines or adenovirus-vectored vaccines (47). The COV-BOOST study (47, 48) conducted in the UK evaluated the safety and immunogenicity of seven different COVID-19 vaccines administered as a third (booster) dose following primary immunization with two doses of either ChAdOx1 nCoV-19 or BNT162b2 for 2878 participants, demonstrating that heterologous booster regimens were safe and often more immunogenic than homologous strategies. Given the ongoing evolution of SARS-CoV-2 variants, the COVID-19 booster should be prioritized for elderly patients with COPD, ideally administered within six months after completion of primary regimen with inactivated vaccines.

This study had several limitations. First, recruitment occurred during the late stage of China’s large-scale primary vaccination campaign with inactivated COVID-19 vaccines, making it difficult to enroll unvaccinated participants as control group and resulting in a relatively small control sample. Second, intending to evaluate the necessity and optimal timing of booster vaccination for high-risk groups to provide evidence for policy maker, booster vaccination was restricted to the target high-risk groups, and the control groups were not eligible to receive the booster, limiting comparative evaluation of booster immunogenicity. Third, as a non-randomized study, potential selection bias and residual confounding related to baseline health status, comorbidities and exposure risk cannot be excluded. Fourth, although this longitudinal study monitored safety outcomes and antibody persistence after vaccination, adverse events and immune durability beyond the follow-up period were not assessed, limiting evaluation of the long-term safety profile and persistence of vaccine-induced immunity in COPD patients. Finally, the cellular immune responses, the immune response to Omicron variants and clinical outcomes including infection, hospitalization and disease severity were not assessed. Despite these limitations, this study provides valuable longitudinal data on the dynamics of three types of antibodies across five time points, as well as evidence on booster timing and safety evaluation among COPD patients, offering important real-world support for vaccination policy decisions in special populations.

## 5 Conclusion

In summary, this study demonstrated that Covilo was safe and well tolerated among COPD patients aged ≥60 years. Although the 2-dose primary series elicited comparable humoral responses, compared to HCs, NAbs levels declined rapidly over time, reflecting limited durability. The homogeneous boosting with Covilo administered after a three-month interval significantly and rapidly increased antibody levels and SCRs, though did not sustain long-term persistence within six months. Given high risk of SARS-CoV-2 infection and severe outcomes in older COPD patients, timely booster administration, preferring to heterologous booster strategies or next-generation innovative vaccines, should be prioritized.

## Data Availability

The raw data supporting the conclusions of this article will be made available by the authors, without undue reservation.
