# Supplementary material for: Safety and antibody responses to inactivated COVID-19 vaccines among elderly patients with COPD: a prospective cohort study
Source: Front Immunol. 2026 Jun 11;17:1840305. doi: 10.3389/fimmu.2026.1840305 (PMC13294153; doi:10.3389/fimmu.2026.1840305)
Supplement: Supplementary file 1 [file Table1.docx]

# Supplementary material

Supplementary Table 1 Incidence of SAEs after each vaccine dose

| SAEs | COPD | | |  | 18–59 HCs | |  | ≥60 HCs | |
| --- | --- | --- | --- | --- | --- | --- | --- | --- | --- |
|  | Dose 1  (n=410) | Dose 2  (n=400) | Dose 3  (n=371) |  | Dose 1  (n=80) | Dose 2  (n=79) |  | Dose 1  (n=108) | Dose 2  (n=106) |
| Fractures and other traumatic injuries | 1 (0.24) | 4 (1.00) | 0 (0.00) |  | 0 (0.00) | 0 (0.00) |  | 0 (0.00) | 0 (0.00) |
| Chronic Obstructive Pulmonary Disease | 1 (0.24) | 3 (0.75) | 0 (0.00) |  | 0 (0.00) | 0 (0.00) |  | 0 (0.00) | 0 (0.00) |
| Cardiovascular/cerebrovascular symptoms | 1 (0.24) | 1 (0.25) | 2 (0.54) |  | 0 (0.00) | 1 (1.27) |  | 0 (0.00) | 1 (1.27) |
| Lower respiratory tract infection | 1 (0.24) | 3 (0.75) | 2 (0.54) |  | 0 (0.00) | 0 (0.00) |  | 0 (0.00) | 0 (0.00) |
| Hepatobiliary diseases | 0 (0.00) | 1 (0.25) | 1 (0.27) |  | 0 (0.00) | 0 (0.00) |  | 0 (0.00) | 0 (0.00) |
| Cervical spondylosis | 0 (0.00) | 1 (0.25) | 0 (0.00) |  | 0 (0.00) | 0 (0.00) |  | 0 (0.00) | 0 (0.00) |
| Chronic renal insufficiency | 0 (0.00) | 0 (0.00) | 0 (0.00) |  | 0 (0.00) | 0 (0.00) |  | 0 (0.00) | 0 (0.00) |
| Total | 3 (0.73) | 9 (2.25) | 6 (1.62) |  | 0 (0.00) | 1 (1.27) |  | 0 (0.00) | 1 (1.27) |

Supplementary Table 2 Comparison of adverse events between groups

| AEs | Dose 1 | | | | |  | Dose 2 | | | | |
| --- | --- | --- | --- | --- | --- | --- | --- | --- | --- | --- | --- |
|  | COPD (n=410) | 18–59 HCs (n=80) | ≥60 HCs (n=108) | χ² / Fisher | *p* value |  | COPD (n=400) | 18–59 HCs (n=79) | ≥60 HCs (n=106) | χ² / Fisher | *p* value |
| **By dose** |  |  |  |  |  |  |  |  |  |  |  |
| Within 3 days |  |  |  |  |  |  |  |  |  |  |  |
| AEs | 26 (6.34) | 1 (1.25) | 2 (1.85) | 6.327 | **0.042#** |  | 9 (2.25) | 0 (0.00) | 1 (0.94) | - | 0.478 |
| Local AEs | 13 (3.17) | 0 (0.00) | 1 (0.93) | - | 0.192 |  | 4 (1.00) | 0 (0.00) | 1 (0.94) | - | 1.000 |
| Systemic AEs | 12 (2.93) | 1 (1.25) | 1 (0.93) | - | 0.539 |  | 5 (1.25) | 0 (0.00) | 0 (0.00) | - | 0.644 |
| Within 7 days |  |  |  |  |  |  |  |  |  |  |  |
| AEs | 26 (6.34) | 1 (1.25) | 2 (1.85) | 6.327 | **0.042#** |  | 10 (2.50) | 0 (0.00) | 1 (0.94) | - | 0.409 |
| Local AEs | 13 (3.17) | 0 (0.00) | 1 (0.93) | - | 0.192 |  | 4 (1.00) | 0 (0.00) | 1 (0.94) | - | 1.000 |
| Systemic AEs | 13 (3.17) | 1 (1.25) | 1 (0.93) | - | 0.429 |  | 6 (1.50) | 0 (0.00) | 0 (0.00) | - | 0.446 |
| During the study period | | | | | | | | | | | |
| AEs | 29 (7.07) | 1 (1.25) | 2 (1.85) | 7.668 | **0.022#** |  | 21 (5.25) | 0 (0.00) | 1 (0.94) | - | **0.016#** |
| Local AEs | 13 (3.17) | 0 (0.00) | 1 (0.93) | - | 0.192 |  | 4 (1.00) | 0 (0.00) | 1 (0.94) | - | 1.000 |
| Systemic AEs | 14 (3.41) | 1 (1.25) | 1 (0.93) | - | 0.390 |  | 6 (1.50) | 0 (0.00) | 0 (0.00) | - | 0.446 |
| **By severity** |  |  |  |  |  |  |  |  |  |  |  |
| Grade 1 |  |  |  |  |  |  |  |  |  |  |  |
| AEs | 19 (4.63) | 0 (0.00) | 2 (1.85) | - | 0.073 |  | 9 (2.25) | 0 (0.00) | 1 (0.94) | - | 0.478 |
| Local AEs | 10 (2.44) | 0 (0.00) | 1 (0.93) | - | 0.407 |  | 5 (1.25) | 0 (0.00) | 0 (0.00) | - | 0.644 |
| Systemic AEs | 10 (2.44) | 0 (0.00) | 1 (0.93) | - | 0.407 |  | 5 (1.25) | 0 (0.00) | 1 (0.94) | - | 1.000 |
| Grade 2 |  |  |  |  |  |  |  |  |  |  |  |
| AEs | 10 (2.44) | 1 (1.25) | 0 (0.00) | - | 0.250 |  | 4 (1.00) | 0 (0.00) | 0 (0.00) | - | 0.768 |
| Local AEs | 3 (0.73) | 0 (0.00) | 0 (0.00) | - | 1.000 |  | 0 (0.00) | 0 (0.00) | 0 (0.00) | / | / |
| Systemic AEs | 5 (1.22) | 1 (1.25) | 0 (0.00) | - | 0.674 |  | 2 (0.50) | 0 (0.00) | 0 (0.00) | - | 1.000 |
| ≥Grade 3 |  |  |  |  |  |  |  |  |  |  |  |
| AEs | 4 (0.98) | 0 (0.00) | 0 (0.00) | - | 0.767 |  | 9 (2.25) | 0 (0.00) | 1 (0.94) | - | 0.478 |
| Local AEs | 0 (0.00) | 0 (0.00) | 0 (0.00) | / | / |  | 0 (0.00) | 0 (0.00) | 0 (0.00) | / | / |
| Systemic AEs | 2 (0.49) | 0 (0.00) | 0 (0.00) | - | 1.000 |  | 0 (0.00) | 0 (0.00) | 0 (0.00) | / | / |

Note: The value represents the χ² value, and the "-" represents the Fisher exact test. # There are no significant differences in pairwise comparisons between the three groups.
